# Health Information Behavior in Parents of Children With Congenital Heart Disease in China: Qualitative Study Through the Lens of Chinese Culture

**DOI:** 10.2196/80784

**Published:** 2025-11-04

**Authors:** Jingran Yang, Fang Ma, Yu Wang, Xingchun Yang, Min Zhou, Yimei Zhang, Ruijie YangLan, Qinglan Li

**Affiliations:** 1 Department of Nursing, The First Affiliated Hospital of Kunming Medical University, No. 295, Xichang Road, Wuhua District, Kunming City, Yunnan Province, 650032, China, 86 871 65324888; 2 Medical Operations Department, Fuwai Yunnan Hospital, Chinese Academy of Medical Sciences, Kunming City, Yunnan Province, China

**Keywords:** congenital heart disease, experiences, health information behavior, information seeking, online, parents, qualitative study

## Abstract

**Background:**

Parents of children with congenital heart disease (CHD) serve as primary caregivers and play a central role in decisions regarding their children’s health care, development, and overall well-being. Their health information behavior directly influences the care decisions and outcomes of their children. In China, the online health information environment is vast but varies in quality, which places a significant information-seeking burden on them in the digital age. Moreover, Chinese cultural backgrounds shape parents’ views, perspectives, and practices related to health information. To date, there have been no studies in China reporting on the experiences of parents of children with CHD concerning their health information behavior.

**Objective:**

The aim of this study was to explore the experiences of health information behavior among parents of children with CHD during the disease journey through the lens of Chinese culture.

**Methods:**

This study used a descriptive phenomenological qualitative method. Face-to-face, semistructured, and in-depth interviews were conducted with parents of children with CHD from March to July 2025 at a tertiary grade A hospital located in Kunming, Yunnan Province, China. Data were collected and managed using the NVivo 12.0 software (QSR International), and thematic analysis was applied to identify and interpret participants’ experiences and perspectives.

**Results:**

A total of 24 parents of children with CHD participated in this study, including 6 fathers and 18 mothers. In total, 6 themes emerged from the data: (1) Looking for health information both online and offline; (2) Seeking health information from professionals and peers as well; (3) Evolving health information needs in the disease journey; (4) Showing diverse attitudes toward health information seeking; (5) Positive and negative feelings during health information behavior process; and (6) Disclosure versus concealment of children’s disease information.

**Conclusions:**

Parents of children with CHD seek health information from both online and offline sources and also combine health information from professionals and peers. Medical institutions should provide authoritative information resources, while regulatory authorities should conduct professional reviews before disseminating health information online to foster a reliable information environment. Additional efforts should focus on utilizing rehabilitation narratives from peer networks, delivering personalized information support tailored to parents’ information-seeking styles and children’s disease stages, and offering training and services to stimulate and cultivate a conscious decision-making process regarding disease disclosure and sharing.

## Introduction

Congenital heart disease (CHD) is defined as any structural or functional abnormality of the heart or great vessels present at birth [1]. As a leading cause of neonatal mortality, CHD accounts for approximately 3% of all neonatal deaths and a substantial 46% of deaths attributable to congenital malformations [2,3]. Globally, it is also recognized as one of the most prevalent birth defects, underscoring its significant public health impact [4]. CHD comprises a heterogeneous spectrum of cardiac malformations, ranging from simple to complex structural defects. Clinically, CHD is classified into mild, moderate, or severe categories based on anatomical abnormalities and their hemodynamic implications. The most frequent types of CHD include simple structural defects such as patent foramen ovale, atrial septal defect, ventricular septal defect, and patent ductus arteriosus, which collectively account for the majority of CHD cases. Other common subtypes encompass bicuspid aortic valve, pulmonary valve stenosis, and complex malformations such as transposition of the great arteries and tetralogy of Fallot [5-7]. Emerging evidence indicates that children with CHD are also at heightened risk of sequelae, such as learning disabilities, attention deficits, and reduced physical endurance, alongside psychosocial challenges encompassing body image disturbances, peer socialization, and emotional dysregulation (eg, anxiety, depression) [8,9]. In addition, the diagnosis, treatment, and long-term management of children with CHD impose comprehensive challenges on parents, encompassing multifaceted stressors such as time commitment, financial burden, and resource coordination [10,11].

Parents of children with CHD are often the primary caregivers and play a central role in making decisions about their child’s health care, development, and overall well-being [12,13]. Their understanding of the disease, available treatments, and potential outcomes can significantly influence the quality of care provided to the child and family well-being [14], which highlights the importance of their health information and related behavior. Health information behavior is a concept derived from information behavior, referring to how people search, screen, use, avoid, or evaluate health-related information in a specific event or context [15,16]. Studies have demonstrated that health information behavior can augment individuals’ health knowledge, influence medical decision-making, and mitigate anxiety and stress [17,18]. A systematic review has also reported a significant association between health information behavior and medication adherence, notably indicating that health information behavior among individuals with AIDS can substantially improve their treatment adherence [19]. These lines of research collectively indicate that parents’ health information behavior enhances their health awareness, strengthens their ability to participate in their child’s disease management, and facilitates their informed medical decision-making, all of which significantly influence the prognosis of children with CHD.

Current studies on the health information behavior experiences among parents of children with CHD remain in its infancy. Previous studies have concentrated on the unmet information needs of parents of children with other chronic conditions [20-22]. While some of these studies recognize the barriers and challenges encountered during information seeking and have explored the sources and mechanisms of information acquisition, few have delved into the full spectrum of caregivers’ information behaviors following diagnosis [23-26], particularly behavioral processes such as how parents select information channels, search for information, evaluate information accuracy, and form attitudes toward disclosing their child’s disease information to others. Moreover, to the best of our knowledge, no study has specifically investigated the unique characteristics and processes that define health information behavior among parents of children with CHD, highlighting a distinct and significant gap in the current literature.

The clinical heterogeneity of CHD directly leads to the diversity of related information and the complexity of management approaches, posing enormous challenges to the information behavior of parents of children with CHD. Furthermore, Chinese culture is characterized by its collectivist nature, where concealment of chronic health conditions is also common [27]. This cultural tendency often translates to parents’ reluctance to both share information about their child’s disease condition and actively seek or openly discuss their child’s health with others, thereby contributing to closedness in their health information behavior. Against this backdrop, the acceleration of digital media and health care digital transformation has significantly enhanced information accessibility, with an increasing proportion of health-related information being acquired via online channels [28]. This further enhances the accessibility of health information, enabling individuals to acquire, process, and utilize information in various ways.

China has a huge number of internet users, accounting for approximately one-fifth of global online users [29]. Although the internet has improved access to a wide range of information, the information on the internet comes from various providers and sources that are difficult to control, which may lead to quality issues and potentially spread biased content based on the interests and purposes involved [30,31]. Hence, it is essential to understand the experiences of health information behavior among parents of children with CHD in today’s eHealth background. An in-depth exploration of the health information behavior patterns among parents of children with CHD is critically significant, as it can inform the improvement of family-centered medical support systems and enhance the quality of health information services. This study aims to address this gap by using qualitative research methods to investigate the health information behavior of parents of children with CHD. The findings will provide a theoretical basis for developing targeted family support strategies and improving health information services, thereby directly enabling health care systems to better serve this vulnerable population.

## Methods

### Study Design

This study adopted descriptive phenomenology to explore the essence of parents’ health information behavior experiences during their children’s CHD journey, as lived by the participants. Phenomenology examines perceptual experiences by bracketing external interpretations (epoché), thereby revealing the nature and essential structure of phenomena through the suspension of preconceptions [32,33]. In this study, the research questions posed are as follows: “How do parents of children with CHD search, screen, use, avoid, or evaluate health information? What are the attitudes and feelings toward health information behavior among parents of children with CHD?” These research questions are consistent with the descriptive phenomenology approach. Additionally, the Consolidated Criteria for Reporting Qualitative Research (COREQ) checklist guided the development, analysis, and reporting of this study (Checklist 1) (see ) [34,35].

### Setting, Participants, and Sampling Method

Yunnan Province, located in southwestern China, has an average altitude exceeding 2000 m and one of the highest incidences of CHD in China; CHD has long been identified as a major pediatric health concern prioritized by local health institutions [36]. Notably, Yunnan Province is characterized by exceptional cultural diversity, underpinned by 25 distinct ethnic minority groups (eg, Yi, Bai, Dai, Hani). This rich multicultural environment not only serves as a unique foundation for cultural research but also gives rise to distinct health beliefs, family care practices, and communication patterns across different ethnic groups [37].

Collectively, these findings suggest that Yunnan may offer a unique research setting for exploring parental health information behavior in the CHD journey.

This study was conducted from March to July 2025 in the Inpatient Department of Cardiology at a tertiary grade A hospital located in Kunming, Yunnan Province, China. This hospital provides medical services for patients from Yunnan Province’s 16 prefectures and surrounding provinces (eg, Sichuan, Guizhou). A purposive sampling method was adopted to identify and recruit eligible participants. Participants were recruited through referrals from clinicians and head nurses, who recommended potential participants meeting the following eligibility criteria to our research team. The inclusion criteria were as follows: (1) parents had a son or daughter with CHD between 0 and 14 years old; (2) parents could understand and express their own experiences and thoughts in Chinese; and (3) parents voluntarily agreed to participate in this study and signed the written informed consent form. The exclusion criteria included (1) parents with a history of mental illness or cognitive impairment, or (2) parents with other serious diseases (eg, malignant tumors, severe organ failure), or (3) parents with hearing or speech impairments, or (4) parents also participating in other research projects, or (5) child with other serious diseases. The purpose of these exclusions was to protect participants’ rights and well-being and ensure data quality. Clinicians and head nurses performed an initial screening of potential participants prior to referral, excluding parents who clearly did not meet the eligibility criteria (eg, parents of children with other serious diseases). Researchers subsequently carried out a systematic screening process during the initial contact with interested parents. This included direct questions regarding parental health status and concurrent participation in other studies. Eligibility assessment was based primarily on parental self-report, supplemented by the researchers’ evaluation of the parents’ ability to comprehend study information and engage in coherent conversation. Clinicians and head nurses recommended 32 parents of children to us, of whom 6 refused to participate due to lack of interest, and another 2 were excluded because they could not accommodate the study schedule. Finally, 24 parents participated in our study.

### Data Collection

From March to July 2025, face-to-face, semistructured, and in-depth interviews were conducted with participants in the hospital’s inpatient wards. A total of 24 parents of children with CHD were interviewed for this study. A female researcher (JRY), who was a current master’s candidate at a medical school with 1 year of cardiology internship experience and had received systematic training in qualitative research, conducted all interviews in Chinese and recorded the participants’ gestures and facial expressions in her notes. The participants and researchers did not know each other, and each interview was conducted only once. After obtaining informed consent from each participant, the interview process was audio-recorded. The interview guide was developed based on the research objectives and relevant literature and then revised through joint discussions by the research team. Before the interviews began, the interviewer explained the concept of health information behavior and the purpose of the study to ensure that participants had the necessary background to provide accurate responses. Firstly, the interviewer explained to the participants that “health information behavior” referred to all kinds of activities in daily life where people looked for, understood, used, and sometimes even avoided health-related information. Then, the interview began by asking about the parents’ general demographic information, including their relationship with the child, age, educational level, place of residence, and the child’s diagnosis. It then transitioned to the main interview outline: (1) What are your experiences and feelings during the process of health information behavior? (2) How do your health information needs change across different stages of the disease? (3) How do you feel about disclosing disease information to others? The full interview guide is detailed in Multimedia Appendix 1. All participants indicated that they understood the concept of health information behavior after explanation, and none asked for further clarification during the interviews. Data collection was concluded upon achieving data saturation, at which point no new concepts emerged from the dataset. Within 24 hours after the interview, the researcher (JRY) performed a verbatim transcription of audio recordings into textual documents. Interviews lasted between 21 and 36 minutes. All interviews were conducted in Chinese, and after data analysis, the quotations used to support the findings were translated from Chinese to English by 2 independent bilingual translators following the forward-back-translation protocol, with the translated versions validated by other researchers to confirm that the original meaning was preserved across languages.

### Data Analysis

Data collection and analysis were simultaneously conducted. Braun and Clarke’s thematic analysis was used for data analysis [38,39]. We chose thematic analysis due to its methodological flexibility. Thematic analysis can identify patterns within and between data on experiences, views, perspectives, behavior, and practices of participants. This method can help to understand what participants think, feel, and do [40]. The thematic analysis was carried out in 6 stages: familiarizing ourselves with the data, generating initial codes, searching for themes, reviewing themes, defining and naming themes, and producing the report [38,41]. First, the researchers (JRY and FM) read each transcript and listened to the audio recording carefully several times to become familiar with the data. Second, researchers (JRY and FM) each extracted significant statements related to the phenomenon of interest and performed independent preliminary coding of the data using the NVivo 12.0 software (QSR International). During this process, they started to notice things of interest and identified a corpus of “instances” of the phenomenon that they were interested in. By doing this, the qualitative data could be reduced, and the researchers would more effectively examine them [42]. Each researcher attached meaning to the significant statement of the data and documented that meaning with a word, symbol, or phrase, which was preliminary coding of the data. After the coding was completed, the researchers compared and discussed the data in order to reach consensus on the final coding. When disagreements arose during the process, YW intervened to resolve the issue through discussion. Third, after the coding was finalized, we started looking for candidate themes. This process was done by recombining and grouping related codes under the same theme until data saturation was achieved [41,43]. Once further cases do not change the coding tree and no new perspectives were found in subsequent interviews, data saturation was achieved. Fourth, 2 other researchers (MZ and YMZ) conducted a rigorous 2-stage review to validate the constructed themes for both thematic validity and reliability. In the first stage (Level 1), they assessed the internal coherence and distinctiveness of coded data within each theme. The second stage (Level 2) involved evaluating theme validity against the entire dataset to ensure comprehensive capture of the data’s meaning. Through this process, overlapping or weakly supported themes were either merged or discarded to enhance the rigor of the thematic analysis. Fifth, clear definitions and names were established for each theme, capturing their essence and relevance to the research question. Lastly, the final synthesis of the results was constructed and confirmed through review by all authors. We did not obtain participant feedback on the findings as participants were discharged from the hospital before the analysis was completed.

### Ethical Considerations

The study was conducted in accordance with the Helsinki Declaration and was approved by the Ethics Committee of the First Affiliated Hospital of Kunming Medical University (Ethics Approval No. 2025L168). Before the interview, written informed consent was obtained from each participant, confirming their voluntary participation in the study. Permission for interview recording was obtained from all participants. Participants had the right to withdraw at any time without providing any reason, and their lack of participation or withdrawal would have no consequences for them. No compensation was offered for participation. The interview content was kept anonymous and confidential and solely used for the purposes of this study. Data were stored on encrypted secure servers, with access restricted exclusively to the researchers. Neither the study nor the supplementary materials contain information that could lead to the identification of individual participants.

## Results

### Participant Characteristics

A total of 24 participants (6 fathers and 18 mothers) were interviewed in this study. The participants ranged in age from 22 to 42 years old. Mothers comprised the majority of the sample (75%, 18/24). In terms of educational level, junior high school was the most common (37.5%, 9/24), followed by college degree (33.3%, 8/24), senior high school (16.7%, 4/24), primary school (8.3%, 2/24), and illiteracy (4.2%, 1/24). Regarding residential location, most participants resided in rural areas (70.8%, 17/24), while 29.2% (7/24) resided in urban settings. The characteristics of participants are shown in Table 1.

Through analysis of qualitative data, 6 salient themes related to the participants’ health information behavior were developed: (1) looking for health information both online and offline; (2) seeking health information from professionals and peers as well; (3) evolving health information needs in the disease journey; (4) showing diverse attitudes toward health information seeking; (5) positive and negative feelings during health information behavior process; and (6) disclosure versus concealment of children’s disease information.

**Table 1.** Characteristics of interviewed parents of children with congenital heart disease (N=24).

ID	Parent’s sex	Parent’s age (y)	The parent’s relationship with the child	The educational level of parent	The residential location of parent	Diagnosis of child
1	Female	31	Mother-daughter	College	Urban	Atrial septal defect
2	Male	32	Father-daughter	College	Rural	Ventricular septal defect
3	Female	33	Mother-son	Junior high school	Rural	Ventricular septal defect
4	Female	42	Mother-son	Junior high school	Rural	Atrial septal defect+patent foramen ovale
5	Female	25	Mother-daughter	College	Urban	Atrial septal defect
6	Female	35	Mother-son	College	Urban	Ventricular septal defect
7	Female	34	Mother-daughter	Junior high school	Rural	Pulmonary valve stenosis
8	Male	35	Father-daughter	College	Urban	Atrial septal defect+ventricular septal defect
9	Male	37	Father-daughter	Illiteracy	Rural	Atrial septal defect
10	Male	38	Father-son	Primary school	Rural	Ventricular septal defect
11	Female	28	Mother-daughter	Junior high school	Rural	Atrial septal defect
12	Female	28	Mother-daughter	Junior high school	Rural	Atrial septal defect+ventricular septal defect
13	Female	22	Mother-son	Senior high school	Rural	Ventricular septal defect
14	Female	31	Mother-son	College	Urban	Ventricular septal defect
15	Female	36	Mother-son	College	Rural	Atrial septal defect
16	Male	30	Father-daughter	Senior high school	Urban	Ventricular septal defect
17	Female	37	Mother-daughter	Junior high school	Rural	Bicuspid aortic valve
18	Female	32	Mother-daughter	Junior high school	Rural	Ventricular septal defect
19	Female	39	Mother-son	Junior high school	Rural	Atrial septal defect
20	Male	33	Father-son	Senior high school	Rural	Atrial septal defect
21	Female	39	Mother-son	Primary school	Rural	Atrial septal defect
22	Female	40	Mother-daughter	Junior high school	Rural	Atrial septal defect
23	Female	33	Mother-daughter	Senior high school	Urban	Ventricular septal defect
24	Female	30	Mother-daughter	College	Rural	Ventricular septal defect

### Theme 1: Looking for Health Information Both Online and Offline

Parents reported primarily obtaining health information through offline consultations with doctors and medically knowledgeable relatives or friends. They viewed these in-person interactions as more credible.

*I consider that the face-to-face consultation with professional medical staff is necessary, especially during the medical decision-making stage, which can help me get accurate information.*

[Participant 5]

Simultaneously, they also relied on online searches due to the internet’s accessibility and breadth of information. While acknowledging the convenience of online resources, parents also noted significant challenges in verifying their accuracy.

*It’s very convenient to go online. By surfing the internet, such as Baidu, Xiaohongshu, and Douyin, we can easily find CHD-related general information.*

[Participant 1]

*There is too much information about CHD on the internet, and some [of it] is controversial…It is hard to distinguish.*

[Participant 5]

### Theme 2: Seeking Health Information From Professionals and Peers as Well

Parents of children with CHD expressed deep trust in medical professionals as their primary source of reliable and authoritative health information, as they recognized their own lack of medical expertise and viewed professionals as credible.

*In fact, for us, we still trust what the doctors said and seek information from them. They are more professional, and the information from medical professionals must be reliable and authoritative.*

[Participant 4]

In the meantime, many parents also actively reached out to other parents of children with CHD to seek learnable experiences and insights. Through these interactions, they not only obtained credible and targeted information support but also found emotional comfort.

*Our neighbor’s child also has CHD. When he was 14 years old, he was diagnosed with CHD by screening, and then he underwent surgery, and now he has recovered. We visit him when [we are] available, and ask him for the information about the disease, how to treat, what should be done in daily care, etc., which helps us a lot.*

[Participant 3]

### Theme 3: Evolving Health Information Needs in the Disease Journey

For some parents, the initial diagnosis of CHD was profoundly shocking news. During this phase, parents prioritize understanding core information about their child’s disease, including disease severity, treatment options, and potential impacts on development. While communication with doctors face-to-face served as the first and most trusted source of information, parents often turned to online platforms immediately afterward to supplement and contextualize the information received from doctors.

*When I found out my child had CHD, the first thing I wanted to know was whether the disease was serious. The doctor told me there was a hole in the child’s heart, and I was scared at that time. I wanted to know how big it was, how serious it was, and what to do next. Afterwards, I went online to find out what a heart hole meant and what might happen next.*

[Participant 5]

*What I wanted to know most was whether it would affect the child’s growth and development and whether it would endanger his life.*

[Participant 3]

Before the surgery, parents’ information needs became more specific and practical, such as the surgical plan, cost details, and preoperative examination indicators. They often turned to online searches to learn about the surgical procedure, but also relied on face-to-face communication with their doctors and nurses to obtain personalized surgical planning, guidance on applying for financial assistance, and interpretation of their child’s preoperative indicators.

*Before the surgery, the attending doctor explained the details we were eager to know, such as the surgical procedure and whether it would be minimally invasive. I also went online to watch videos about how the surgery is done.*

[Participant 6]

*Cost of surgery was another concern. There were some grants that could cover the cost; we wanted the information from doctors or nurses about how to apply [for] the grant.*

[Participant 2]

*We would pay attention to the child’s various examination indicators before surgery, being afraid that if the indicators were not optimal and the child couldn’t undergo surgery.*

[Participant 3]

After surgery, parents of children with CHD had an urgent need for recovery and rehabilitation information. When seeking immediate information such as their child’s current status in the intensive care unit (ICU), they strongly preferred direct communication with health care professionals.

*After the surgery, I was afraid that she would be crying alone in the ICU, because I couldn’t accompany her. I wanted to know her condition. I asked the nurses every day for details.*

[Participant 5]

*A few days after the operation, he had a potassium deficiency. We went directly to ask the nurse what he should eat to supplement it. The nurse was able to give us accurate recommendations based on his specific condition.*

[Participant 6]

Some parents turned to online channels to access practical nursing tips for wound care and scar repair after discharge.

*I searched online about how to reduce surgery scars and what creams to use.*

[Participant 22]

### Theme 4: Showing Diverse Attitudes Toward Health Information Seeking

Some parents considered CHD-related information to be extremely important and made proactive and exhaustive efforts to obtain it through diverse channels, including online platforms, their social networks, and health care professionals.

*Since my child got sick, I couldn’t sleep at night and felt completely at a loss. The only thing I could do was seek information everywhere—searching online, asking relatives and friends, and consulting doctors at different hospitals. I was desperate to gather every piece of information; afraid I might miss something critical.*

[Participant 12]

Some participants reflected that they would take the initiative to seek relevant information to help their children achieve rehabilitation, but they automatically filtered out negative information to reduce anxiety.

*I would search for treatment plans and nursing tips for CHD online via my phone, but deliberately avoid negative information, which may likely affect my mindset and unnecessarily increase anxiety.*

[Participant 1]

However, some parents did not take the initiative to seek health-related information themselves; instead, these parents placed their trust entirely in doctors and preferred to rely on them for all health-related information and guidance.

*For the treatment of children, I mostly follow the advice of doctors and rarely take the initiative to learn or search on my phone for how to deal with diseases. I think it’s useless to read too much.*

[Participant 4]

### Theme 5: Positive and Negative Feelings During Health Information Behavior Process

Parents of children with CHD experienced a range of positive and negative feelings throughout the process of their health information behavior. Clinicians translating complex medical terminology into accessible language reduced parents’ confusion and enhanced their sense of understanding.

*When the doctor talked about ventricular septal defect, he explained it to us in detail and used a metaphor (the heart was like a house, and there was a hole in the wall, then we needed to repair the hole in the house). We could understand it easily. That was great.*

[Participant 3]

Beyond clinical communications, peer-shared online resources (eg, rehabilitation narratives and practical hospitalization guides) enhanced treatment confidence and reduced anxiety.

*Some positive cases online have given me great confidence. I just saw online that after the surgery, the children with CHD had good physical growth and good exercise ability, so I have hope and expectations.*

[Participant 5]

In contrast to these positive feelings, parents also faced significant challenges that triggered negative feelings. Many parents faced persistent difficulties in comprehending specialized jargon, which created immediate barriers to understanding and left them feeling powerless.

*The biggest difficulty is not understanding. We cannot understand what the doctors are talking, that’s terrible.*

[Participant 3]

Exposure to distressing online case reports also exacerbated parental anxiety about their children’s prognosis.

*I have watched a negative video about a child who underwent two surgeries for heart problems and unfortunately passed away at home after the second surgery. This makes me worried that my own child might have to go through multiple surgeries like that child in the video but end up with nothing, which inevitably increases my anxiety.*

[Participant 3]

Furthermore, limited access to quality offline medical resources added to parents’ sense of urgency and helplessness.

*It’s difficult to get an expert appointment, and the appointment has been fully booked for almost two weeks. I’m worried that I won’t be able to register offline and it will be troublesome to seek medical attention.*

[Participant 5]

Additionally, a lack of clear and timely communication from health care providers during clinical interactions was a potent source of anxiety.

*When I was doing a cardiac ultrasound, I came out after finishing one in the consultation room, but the doctors asked me to wait without saying the reason. I was very anxious and didn’t know if I needed to check again.*

[Participant 6]

### Theme 6: Disclosure Versus Concealment of Children’s Disease Information

Many parents actively disclosed their children’s disease information based on a sense of familial obligation and the need for support. They believed that concealing the disease information from family members would damage trust and miss out on potential support.

*I think we must tell our family about the child’s condition—after all, we’re family. If we hide it, and they find out later, they will definitely ask why we didn’t say anything and might even blame us… telling them might also get their support.*

[Participant 9]

Beyond familial duty, many parents actively disclosed their children’s disease information to relatives and friends to seek emotional comfort and medical resources.

*When I found out about my child’s illness, I was in a bad mood, so I confided in my family, and they were able to comfort me.*

[Participant 1]

*I told my relatives and some friends about my child’s CHD diagnosis and asked if they had encountered similar situations or knew any good medical resources to recommend.*

[Participant 10]

In addition, some parents stated that they were willing to share the experience of accompanying their children in the fight against the disease with others in need, so as to pass on experience and strength.

*I’d like to share my child’s experience of fighting the disease with other parents. I not only want to give them some advice but also hope they can learn from our past experiences to avoid making the same mistakes.*

[Participant 8]

In certain situations, disclosure was compelled by external requirements or situational constraints.

For instance, parents disclosed their child’s CHD to schools to ensure the child’s safety and secure necessary school-provided support in case of medical emergencies.

*The teacher will come to visit our child before he starts kindergarten, and they’ll ask if there are any special circumstances that need their attention. We thought it’s better to let the teacher know in case of an emergency, so they can pay extra attention.*

[Participant 6]

Another reason for disease disclosure stemmed from workplace demands. When parents required frequent leave to accompany their child to medical appointments (such as regular checkups or treatment sessions), they often felt compelled to disclose the child’s disease information to employers or colleagues in order to provide a legitimate justification for these absences.

*I had to take my child for surgery and then asked for leave.*

[Participant 5]

Conversely, some parents opted for concealment of the child’s disease information due to concerns about privacy, potential peer rejection, and the social stigma associated with CHD. They were concerned that disclosing the child’s disease information might have a negative impact on the child’s social development and long-term well-being.

*This is a highly private matter. I worry that others may misunderstand CHD, assuming that affected children are severely ill or fundamentally different from their peers. Such misconceptions could lead to social exclusion, which might negatively impact the child’s emotional and social development.*

[Participant 12]

## Discussion

### Principal Findings

To our knowledge, this is the first qualitative study in China to explore the experiences of parents of children with CHD during the disease journey. A total of 6 key themes were identified in this study. (1) Parents look for health information through both online and offline channels. (2) Parents consult both professionals and peers for information support. (3) Parents’ information needs vary significantly across different stages of the disease journey. (4) Parents’ attitudes toward information seeking differ considerably among parents. (5) Parents’ experiences with their health information behavior included both positive and negative aspects. (6) Parents hold diverse attitudes regarding the disclosure of their child’s disease information. These findings underscore the importance of recognizing the unique characteristics of this population’s information behavior.

This calls for concerted efforts to improve the online information environment while also providing personalized guidance tailored to parents’ information-seeking styles and their child’s disease phase.

The study found that parents of children with CHD adopted a complementary strategy of combining offline channels and online open platforms when seeking health information. McCosker et al [44] pointed out that professional knowledge from general practitioners is more valued than online health information. Wainstein et al [45] also reported that 88% of Australians stated they trust their doctors more than the internet. Previous studies have noted that during the process of searching for health information online, users usually retrieve a large amount of information of varying quality, which makes it difficult for users to discern the authenticity of the information [46,47]. This is also evidenced by Gibson’s study on the information behavior of parents of individuals with disabilities, in which trust in veracity and currency of information found online was a concern for parents seeking information online [48]. Moreover, compared with Western countries, the issue of low-quality online health information appears to be particularly severe in Asian countries [49]. Therefore, parents of children with CHD may prioritize obtaining information through offline, face-to-face communication with health care professionals. However, limited access to quality offline medical resources might lead to information poverty among these parents, which is often a result of systemic failure of information systems to meet the needs of marginalized groups; hence, emphasis should be placed on the development and improvement of the systems [48]. We call on online authors, internet platforms, and regulatory authorities to conduct professional reviews before disseminating health information, so as to ensure the accuracy and reliability of information on the internet [50].

Consistent with other research, we found that parents seek health information from both medical professionals and peers [20]. Professional guidance from doctors can provide direct, targeted, and effective disease treatment, while “informal experiences” shared by peers can offer more direct care experiences and emotional support [51]. However, unlike another study that suggests peer support only plays a secondary role [44], the participants in this study regarded the information provided by peers as equally important, especially in terms of gaining experiences and coping strategies. Chinese familism culture places the family at the center of the social structure, emphasizing that individuals cannot achieve an ideal life independently of the organization and viewing society as an “extended family” [52-54]. This cultural framework may shape how parents of children with illnesses engage with health information [55]. Specifically, it motivates them to proactively seek support and information from peers within this “extended family” (eg, other parents of children with CHD in support groups or community networks).

The results of our study indicate that parents of children with CHD have varying information needs at different disease stages. A study shows that providing sufficient preoperative information to parents can significantly reduce their anxiety levels and uncertainty [56]. Providing information to parents through established educational programs before surgery can alleviate their fears and anxiety, enhance their understanding of the surgical process, and enable them to better prepare to participate in different stages of their child’s surgical care. Notably, after surgery, parents of children with CHD often expressed a strong desire to stay informed about their child’s condition in the ICU. Temporary separation from their child tends to heighten feelings of uncertainty and trigger separation anxiety [57]. This underscores the need to implement a flexible ICU visitation policy, which can help alleviate parents’ anxiety and fear stemming from the unknowns of the ICU [58]. Guided by the chronic illness trajectory model [59], health care providers should offer personalized consultations and information support for parents tailored to their child’s disease phase, such as providing basic disease knowledge during diagnosis; interpreting treatment plans, clarifying medical policies, and outlining risk management strategies pre-surgery; and supplying rehabilitation guidance post-surgery.

Our study found that the attitudes toward information seeking differed considerably among parents of children with CHD. Some parents made a proactive and exhaustive effort to obtain health information regarding their children’s conditions. This might be explained by Miller’s blunting theory and the Cognitive-Social Health Information Processing model. According to the Cognitive-Social Health Information Processing model, individuals can be categorized into 2 primary types in terms of medical information seeking: monitoring information-seeking styles (also known as monitors) and blunting information-seeking styles (also known as blunters) [60]. Monitors actively seek out health-relevant information, whereas blunters tend to avoid such information. In our study, parents with a monitoring information-seeking style proactively sought out large amounts of health-related information, which may help reduce their sense of uncertainty [61,62]. We did not find any parents who actively avoided health-related information. This might be explained by the fact that parents consider themselves highly responsible for the care of their children. Traditional Chinese culture emphasizes that “parents who love their children shall plan for their long-term well-being” [63], highlighting the “responsibility of upbringing” as a core ethical obligation and duty of parents, which reinforces parents’ inescapable responsibility for their children’s health. Parents’ avoidance of health-related information would be regarded as dereliction of duty. Our study found that some parents only followed doctors’ advice without proactively seeking any health information. This may be associated with Chinese philosophies influenced by Buddhism, which emphasize fate’s role in health and view death as both a natural law and a continuation of life [64,65]. Such beliefs may make parents feel their efforts to address their child’s illness are useless, as suffering and recovery are both governed by fate. Another factor is Chinese patients’ deep respect for medical staff. In doctor-patient interactions, they often take a subordinate role and consider that professionals are fully capable of managing health issues [64,66]. Additionally, a doctor being in a position of power may result in parents’ passive information behavior, who may resort to defensive behaviors such as “waiting” for information [48]. We found out that some parents would filter out negative information, which is also evidenced by Zhuo et al [67]. This may be due to the fact that exposure to words related to negative or risky information may frighten them and trigger feelings of depression and anxiety. The diverse attitudes of parents when seeking health information suggest that when providing information to parents, we need to consider parents’ information-seeking styles and take into account cultural and individual differences to offer targeted support.

Parents’ experiences with their health information behavior included both positive and negative aspects. A key difficulty they faced in this process was struggling to understand professional terminology. Aligned with the findings of this study, Mentrup et al [68] noted in his qualitative synthesis research that participants expressed a preference for health information to be delivered in accessible language rather than through complex medical terminology. The use of interactive visualizations such as visual infographics can present complex information in a simple manner and help patients better understand it [69,70]. Our study showed that recovery narratives shared by peers were a positive experience for parents, which could strengthen parents’ confidence in their children’s recovery. This may be explained by the following mechanisms: (1) recipients being able to obtain useful information from the experiences of others facing similar health issues and (2) individuals feeling supported and empowered when recognizing that others have encountered and overcome comparable challenges [71,72]. Therefore, it is suggested to transform abstract medical knowledge into patient-perceivable scenarios and utilize interactive visualizations and recovery narratives to enhance parents’ positive experiences in the process of health information behavior.

We found that some parents regarded disclosing their children’s disease information to family members as an inescapable ethical responsibility. Confucianism emphasizes the central role of the family in health care decision-making; to a certain extent, the importance of family informed consent even surpasses that of individual informed consent [73]. The joint participation of all family members ensures the maintenance of sound family relationships [74]. Some parents indicated that they would be willing to share their children’s experiences of fighting the disease with others in need, which once again confirms the importance and necessity of peer support networks. This can also be explained by the mechanism of narratives and the fact that people are more willing to disseminate knowledge, experiences, and insights through sharing [75,76]. However, some parents chose to conceal their child’s illness from others. Privacy concerns typically influence the extent to which individuals are willing to share sensitive information [77,78]. Another study also notes that the stigma associated with illness and fear of negative reactions may lead to reluctance to disclose illness [79]. Policymakers and health care providers should also increase public understanding of CHD by launching targeted public awareness programs and disseminating reliable, accessible CHD-related information, thereby reducing the stigma associated with CHD in society [80]. In the future, we can provide training and services aimed at stimulating and cultivating a conscious decision-making process regarding disease disclosure and sharing.

### Strengths and Limitations

This study explored the experiences of health information behavior among parents of children with CHD within the Chinese cultural and contextual framework. It addresses the gap in existing data on the health information behavior experiences of this population. However, several limitations should be considered. First, this study excluded parents with mental illness, a group that constitutes a significant portion of the adult population, as well as children with comorbid diagnoses. This exclusion criterion limits the transferability of our findings, as the perspectives on health information behaviors of these specific sub-groups were not captured. Future research could explicitly include parents with mental illness and explore the health information behavior experiences of families of children with both CHD and comorbidities to fill this gap. Second, this study was conducted in the context of CHD, so the results may not be readily generalizable to parents of children with other illnesses. Future studies could explore this topic across different diseases. Third, all participants were recruited from a single hospital in 1 region of China, which may further limit the generalizability of the results. Caution is therefore advised when extrapolating the findings to other settings or populations. Lastly, this study was conducted within the Chinese cultural context, and different cultural environments may lead to diverse experiences and perspectives. Future studies could be extended to multiple hospitals across regions and diverse ethnic groups to enhance the generalizability and depth of the findings.

### Conclusions

This study reported on the experiences of parents of children with CHD regarding their health information behavior during the disease journey under the Chinese background. In terms of information-seeking sources, parents generally seek information from both online and offline sources and also combine health information from professionals and peers. We call on online authors, internet platforms, and regulatory authorities to conduct professional reviews before disseminating health information, so as to ensure the accuracy and reliability of information on the internet. We can explore and utilize rehabilitation narrative resources within peer support networks to enhance the positive experiences of parents’ health information behavior. Health care providers should take into account parents’ information-seeking styles and their cultural background characteristics when providing personalized consultations and information support, supplemented by information systems tailored to different disease stages. Parents of children with CHD hold varying attitudes toward the disclosure of disease information. Policymakers and health care providers should increase public understanding of CHD by providing appropriate educational programs to reduce the stigma associated with CHD in society and offering training and services to stimulate and cultivate a conscious decision-making process regarding disease disclosure and sharing.

## Supplementary material

10.2196/80784 Multimedia Appendix 1 Interview guide.

10.2196/80784 Checklist 1 COREQ checklist

## Abbreviations

CHD: congenital heart disease
COREQ: Consolidated Criteria for Reporting Qualitative Research
ICU: intensive care unit
