# Supplementary material for: Health Information Behavior in Parents of Children With Congenital Heart Disease in China: Qualitative Study Through the Lens of Chinese Culture
Source: J Med Internet Res. 2025 Nov 4;27:e80784. doi: 10.2196/80784 (PMC12585002; doi:10.2196/80784)
Supplement: Multimedia Appendix 1 [file jmir-v27-e80784-s001.docx]

**Interview guide**

| **Introduction​**  ⦁ Provide an introduction with the background of the study, the aims, and the structure of the interview.​  ⦁ Explain the concept of health information behavior to ensure that participants have the necessary background to provide accurate responses. The interviewer explains to the participants that “health information behavior” refers to all kinds of activities in daily life in which people look for, understand, use, and sometimes even avoid health-related information. | | |
| --- | --- | --- |
| Question no. | Main question | Probe (Specific, parent-friendly question) |
| 1 | What are the sources and channels of your health information? | ⦁Where do you looking for information about your child’s heart condition? |
| 2 | Please talk about your views on health information? | ⦁In general, how do you think of the information that’s available on your child’s heart condition?​ |
| 3 | What are your experiences and feelings when seeking, avoiding, evaluating, and utilizing health information? | ⦁Could you tell me about your own experiences when looking for child’s heart information?​  ⦁What was it like trying to find the information that you needed?​  ⦁Were there ever times you decided to stop looking for information or avoid certain details, and why did you do that?​  ⦁How do you usually decide if the information you find is trustworthy and reliable?​  ⦁ Once you find information, how do you use it to help you?​ |
| 4 | How do your health information needs change across different stages of the disease? | ⦁ From the time your child was first diagnosed until now, what were the different information needs in different time period of the disease journey.  ⦁ What did you need to know most back then, and what are you most concerned about now?​ |
| 6 | How do you think of sharing disease information with others? | ⦁ Do you share the information of the disease with others? How do you think of it?​  ⦁ Are there any reasons you might sometimes decide not to share it?​ |
| 7 | What suggestions or expectations do you have for a better experience with information behavior? | ⦁Thinking about your entire experience of looking for and using information about CHD, what could be better? ⦁What kind of support or resources would have made this easier for you? |
| **Closing​**  ⦁ Do you have any other experiences or feelings about this topic that you would like to add?​  ⦁ Thank you for your participation, and this interview is now closed. | | |
